# Cost-effectiveness of therapeutic drug monitoring of perampanel for pediatrics with epilepsy: real-world data

**DOI:** 10.3389/fphar.2025.1549069

**Published:** 2025-06-30

**Authors:** Yani Hu, Pu Miao, Shunan Chen, Fengqian Mao, Suhong Wang, Jianhua Feng, Jie Chen, Lingyan Yu, Haibin Dai

**Affiliations:** ^1^ Department of Pharmacy, School of Medicine, Second Affiliated Hospital of Zhejiang University, Hangzhou, Zhejiang, China; ^2^ Department of Pediatrics, School of Medicine, Second Affiliated Hospital of Zhejiang University, Hangzhou, Zhejiang, China

**Keywords:** therapeutic drug monitoring, cost-effectiveness, pediatrics, efficacy, epilepsy, perampanel (PER)

## Abstract

**Aims:**

Increased plasma concentration of perampanel (PER) is associated with reduction in seizure frequency. It is unclear how much better therapeutic drug monitoring (TDM) is than without TDM in pediatric using PER. This study aims to estimate the effectiveness and cost-effectiveness of TDM of PER in children with epilepsy.

**Methods:**

An observational study was conducted to compare clinical and economic outcomes between the TDM and non-TDM groups. We used a Markov model to evaluate the cost-effectiveness of TDM from a healthcare system perspective based on data from an observation cohort study that included four health states. High cost-effectiveness thresholds were defined as < $12,814. One-way, probabilistic sensitivity analyses and scenario analyses were conducted to explore uncertainty.

**Results:**

TDM of the PER improved the 1-year seizure-free rate from 16.7% to 48.1% and the >50% reduction in seizure frequency from 58.3% to 83.3% in children with epilepsy (P = 0.035). The TDM of the PER is highly cost-effective, with an ICER of $732.90 per QALY gained (79.11% probability of being highly cost-effective). TDM was dominant, with an increased QALY of 0.86 and a decreased cost of $1,586.07 in children with newly diagnosed epilepsy. The number of days of hospitalization in the no response state, which is related to the cost of hospitalization, is the largest influencing factor.

**Conclusion:**

TDM of PER could reduce the seizure frequency and is cost-effective for children with epilepsy. TDM of the PER in newly diagnosed epilepsy patients is strongly dominant because of its improvement in efficacy and reduction in cost.

## Introduction

Perampanel (PER) is a newer anti-seizure medication (ASM) that has received approval for use as adjunctive therapy or monotherapy in adults and children aged ≥4 years old with focal-onset seizures, with or without focal to bilateral tonic‒clonic seizures (Krauss et al., 2012; Yamamoto et al., 2020). PER is metabolized in the liver primarily via CYP3A4.

Therapeutic drug monitoring (TDM) measures therapeutic drug concentrations in the biofluid of patients to guide dosage adjustment (Meneghello et al., 2018). Studies have shown a positive correlation between the plasma concentration of PER and the improvement of epilepsy control, indicating that as the plasma concentration increases, the frequency of seizures decreases (Gidal et al., 2013; Franco et al., 2016). A linear relationship was also observed between the PER concentration and dosage in adults and children (Qu et al., 2023; Steinhoff et al., 2019).

Some special situations, such as combinations of other drugs, elderly individuals, children, and patients with special disease states, still require the monitoring of PER. Compared to that of PER combined with enzyme-induced ASMs, the efficacy of PER combined with non-enzyme-induced ASMs was better (Hou et al., 2023). In combination with phenytoin (PHT), carbamazepine (CBZ), oxcarbazepine (OXC), and topiramate (TPM) enhance metabolism and decrease plasma concentrations of PER, while ketoconazole inhibits metabolism and increases plasma concentrations (Patsalos et al., 2018). The plasma clearance of PER in children is significantly greater than that in adults and decreases gradually with age (Li Y. et al., 2022). However, the deceasing course is characterized by interindividual variability (Italiano and Perucca, 2013). Furthermore, PER has not been proven to be useful for children aged 1–4 years, and a suitable dosage needs further research. Most studies have shown that adverse reactions are not related to the concentration of PER (Gidal et al., 2013; Hou et al., 2023; Li Y. et al., 2022). However, most studies on TDM of PER are single cohort, and it is unclear how much better TDM is than without TDM in pediatric using PER to control seizure.

In this study, we first evaluate the effectiveness of TDM of PER in real world and then use real-world data to investigate the cost-effectiveness of TDM of PER.

## Methods

### Study cohorts

Our study population was based on an observation cohort study from 1 October 2020, to 30 September 2023. All patients included were diagnosed with epilepsy (Fisher et al., 2014), were <18 years old, had never used PER before, and were followed up for at least 1 year. Patients were from the second Affiliated Hospital of Zhejiang University School of Medicine, which is a comprehensive hospital with six campuses providing medical services across multiple regions. Patients who had poor compliance, who were transferred to other non-ASM therapies during follow-up period (electrical nerve stimulation, surgery, or a ketogenic diet) were excluded. Compliance was evaluated by Simplified Medication Adherence Questionnaire (Ma et al., 2019). The missing data was addressed through multiple imputation.

Patients who received TDM are in the TDM group, while those who did not are in the non-TDM group. The initial dose of PER is 2 mg/day. The decision for patients initially prescribed PER to receive TDM was random. The dosage adjustment of PER in TDM group aimed to maintain a benchmark concentration when optimal control of seizures was achieved. Dosage in TDM group was adjusted according to the TDM results of PER and symptom: 1. If patients do not reach the therapeutic range without seizure free, increase the PER dose based on TDM results; 2. If patients are within the therapeutic range with seizure free, maintain the dosage; 3. If patients are within the therapeutic range without seizure free, increase the dosage by 2 mg/day each time; 4. If patients are beyond the therapeutic range with seizure free, decrease the dosage according to the TDM results; 5. If patients are beyond the therapeutic range without seizure free, consider other therapies. The therapeutic range of PER is 100–1000 ng/mL (Yu et al., 2023). The plasma concentration of PER was routinely monitored once a month and tested again 1 week after dose adjustment or immediately after adverse reactions occur. All TDM samples were detected by liquid chromatography-mass spectrometry (LC-MS) and were through concentration of PER. If patients in the non TDM group still experience seizures, the dose will be increased by 2 mg/day every 2 weeks until there are no seizures or the patient cannot tolerate it, with a maximum dose of 12 mg/day.

Patient characteristics, epilepsy type (classification based on seizure semiology, electroencephalography (EEG) findings and magnetic resonance imaging), seizure frequency, ASM combination (drug dosage, frequency and adherence), family history and TDM results were collected from the first PER prescription to the end of follow-up. We followed up patients every 2 weeks for the first month, once a month for next 2 months and once every 3 months for rest of the time until lasted for 1 year. In addition, the direct costs of each patient during the follow-up period were collected for economic model parameter input, including ASM therapy, routine outpatient monitoring, hospitalization, TDM. The data were obtained from the hospital medical system. The study was conducted according to the guidelines of the Declaration of Helsinki, and approved by the ethics committee of Second Affiliated Hospital of Zhejiang University, School of Medicine (IR2023385, 2024/1/22). All included patients provided written informed consent.

The primary clinical outcome was the 6-month and 12-month response rate (percentage of seizure frequency decreases ≥50%). The secondary clinical outcome was the 12-month complete response rate, that is, the seizure freedom rate (the percentage of 100% reduction in seizure frequency) (Krauss et al., 2020).

### Economic model

We constructed a decision model to estimate the cost-effectiveness of TDM in pediatric patients with epilepsy. The decision model adopted the perspective of the Chinese health system. A 15-year horizon was chosen to reflect chronic epilepsy, with a cycle length of 6 months to match the primary clinical outcome of the observation study. Costs and outcomes were discounted at 5% annually, in line with the Chinese Pharmacoeconomic Evaluation Guidelines 2020 (Chinese Pharmacoeconomic Evaluation Guidelines, 2020).

### Model structure

A Markov model was developed to emulate epilepsy pediatric transition among four health states according to seizure frequency reduction and cessation of PER therapy in individuals: no response (<50% reduction in seizure frequency), response (≥50% to <100% reduction in seizure frequency), seizure freedom (100% reduction in seizure frequency), and cessation of PER. The model structure was consistent with our real-world data. One hundred percent of children who adopted PER entered the model in the “no response” health state and could move to other health states.

**FIGURE 1:**
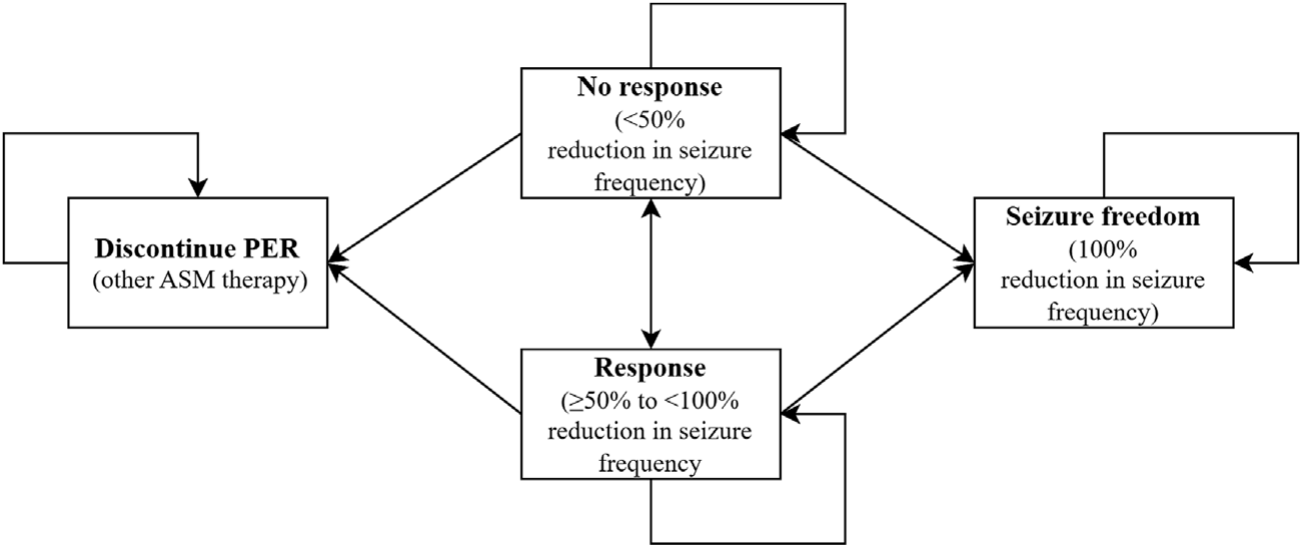
Markov model structure. ASM, Anti-seizure medication.

Patients could discontinue PER therapy in any health state unless they achieved seizure freedom and could convert to other ASM therapies that did not require TDM of the PER. Therefore, patients will remain in the discontinued PER state until the Markov cohort ends, but direct medical costs accumulate. Based on real-world data, our model provides 13 possible alternative ASMs, including CBZ, clonazepam (CLZ), clobazam (CLB), lacosamide (LCS), levetiracetam (LEV), lamotrigine (LTG), OXC, phenobarbital (PB), PER, TPM, valproate (VPA), vigabatrin (VGB), nitrazepam, and zonisamide (ZNS). The model structure is displayed in Figure 1.

### Model input parameters

The real-world data were used to parameterize the transition probability for PER with or without TDM (Table 2). The first cycle transition probability parametrizes movement among on-treatment people for the first 6-month phase. As the follow-up only lasted for 1 year, we assumed that the transition probability of the second cycle onward is the same based on data from the 6th to 12th month phases. Ordinal logistic regression was used to calculate the OR of the TDM group relative to the non-TDM group as clinical outcomes were classified into three levels (uncontrol, response, and seizure free). Real-world estimates of adverse reactions were not available in this study. As such, we captured the incidence rate of adverse reactions from the literature and consolidated the data (Qu et al., 2023; Li Y. et al., 2022; Lin et al., 2018). The incidence rate of adverse reactions in TDM group is lower than non-TDM group. For utility, we referred to a mapping study to determine the short-form six-dimensional (SF-6D) score based on seizure frequency (Flint et al., 2023). The seizure frequency in 28 days from real-word data was applied in regression to generate utility for cost-effectiveness analysis. Adverse reactions may occur in each cycle, causing a decrease in the utility value (Laskier et al., 2023). Since few and mild adverse reactions to PER have been reported, we considered the following four mild to moderate adverse reactions: dizziness, somnolence, irritability, and ataxia (Li D. et al., 2022; Rugg-Gunn, 2014).

Cost categories consisted of ASM therapy, routine outpatient monitoring, hospitalization, TDM, and adverse reactions. Costs from real-world studies were all inflated to the year 2022, and the average inflation rate is 1.48% (CNY Inflation Calculator, 2024).

The costs of ASM therapy were spilt to those of PER and other combined ASMs. The PER cost was calculated by the average daily dose of PER (mg/d) multiplied by the cycle length and average cost per unit dose (RMB/mg). Other combined ASM therapy costs were estimated as weighted average costs according to the proportion of patients who received the combined ASM, the average daily dose of the combined ASM and the associated pack price. The average daily dose and proportion of combinations with other ASMs captured from real-world data are different for each health state.

The TDM cost was multiplied by the number of TDM and unit price of TDM. Hospitalization costs were determined by the probability of hospitalization, the number of days of hospitalization, and the average daily cost of hospitalization. Patients received routine outpatient monitoring, so the number of outpatients, test cost, examination cost and number of consultations were calculated. For patients who discontinued PER, the ASM cost was estimated by the weighted average cost according to the probability of switching to another drug combined with other drugs. The unit cost of adverse reactions was obtained from the China Health Statistics Yearbook (2022).

### Cost-effectiveness analysis

The base case analysis was represented by the incremental cost-effectiveness ratio (ICER), an indicator of incremental cost on additional quality-adjusted life years (QALY) gained by TDM. Cost-effectiveness thresholds of <12,814 US dollars (Chinese GDP per capita in 2022), 12,814 to <38,442 US dollars (three times the GDP per capita) and ≥38,442 US dollars per QALY gained were defined as highly, intermediately or not cost-effective, respectively (Anderson et al., 2014). The willingness-to-pay (WTP) threshold for epilepsy is similar to GDP per capita, not 3 times GDP per capita, as set by the guidelines (Gao et al., 2015). In addition, research suggests that the willingness to pay varies by disease (Kvamme et al., 2010). Therefore, we used GDP per capita as the threshold, referring to how to set the WTP threshold for cardiovascular disease (Anderson et al., 2014). All costs in RMB have been converted to US dollars using the 2022 exchange rate. 1 US dollar = 6.73 RMB (Exchange Rate, 2024).

Our data on efficacy, cost, and other relevant factors are derived from real-world study. To minimize the bias introduced by the research, one-way sensitivity analysis was performed to identify the impact of input parameters, such as the cost of PER and other ASMs, the OR of the non-TDM group relative to the TDM group, the TDM cost, the probability of hospitalization and adverse reactions, and health state utility. The 95% confidence interval (CI) was calculated for each parameter that varied in the one-way sensitivity analysis. When the 95% CIs were unavailable, a standard error of 20% of the mean value was assumed (Drummond et al., 2005). We conducted probabilistic sensitivity analysis using 10,000 Monte Carlo simulations to explore the parameter joint uncertainty. We assigned gamma, beta and normal distributions for cost, probability and utility respectively (Briggs et al., 2006; Briggs et al., 2001). Ratio was assigned lognormal distribution, and the disutility value was assigned gamma distribution (Briggs et al., 2006; Drummond and McGuire, 2001). Table 2 details the distributions for parameters. The probability of being cost-effective was calculated between 12,814 and 38,442 US dollars per QALY gained.

Scenario analyses were performed as follows: (Krauss et al., 2012): all patients were newly diagnosed with epilepsy; (Yamamoto et al., 2020); TDM-guided dosage adjustment lasted for only 1 year, with no effect gained in the next cycle because frequent dose adjustments usually occurred within 1 year of beginning the ASM; (Meneghello et al., 2018); there was a 1-year time horizon, 5-year time horizon and 10-year time horizon; and (Gidal et al., 2013) all patients had refractory epilepsy.

## Results

### Cohort characteristics, outcomes and inputs

**FIGURE 2:**
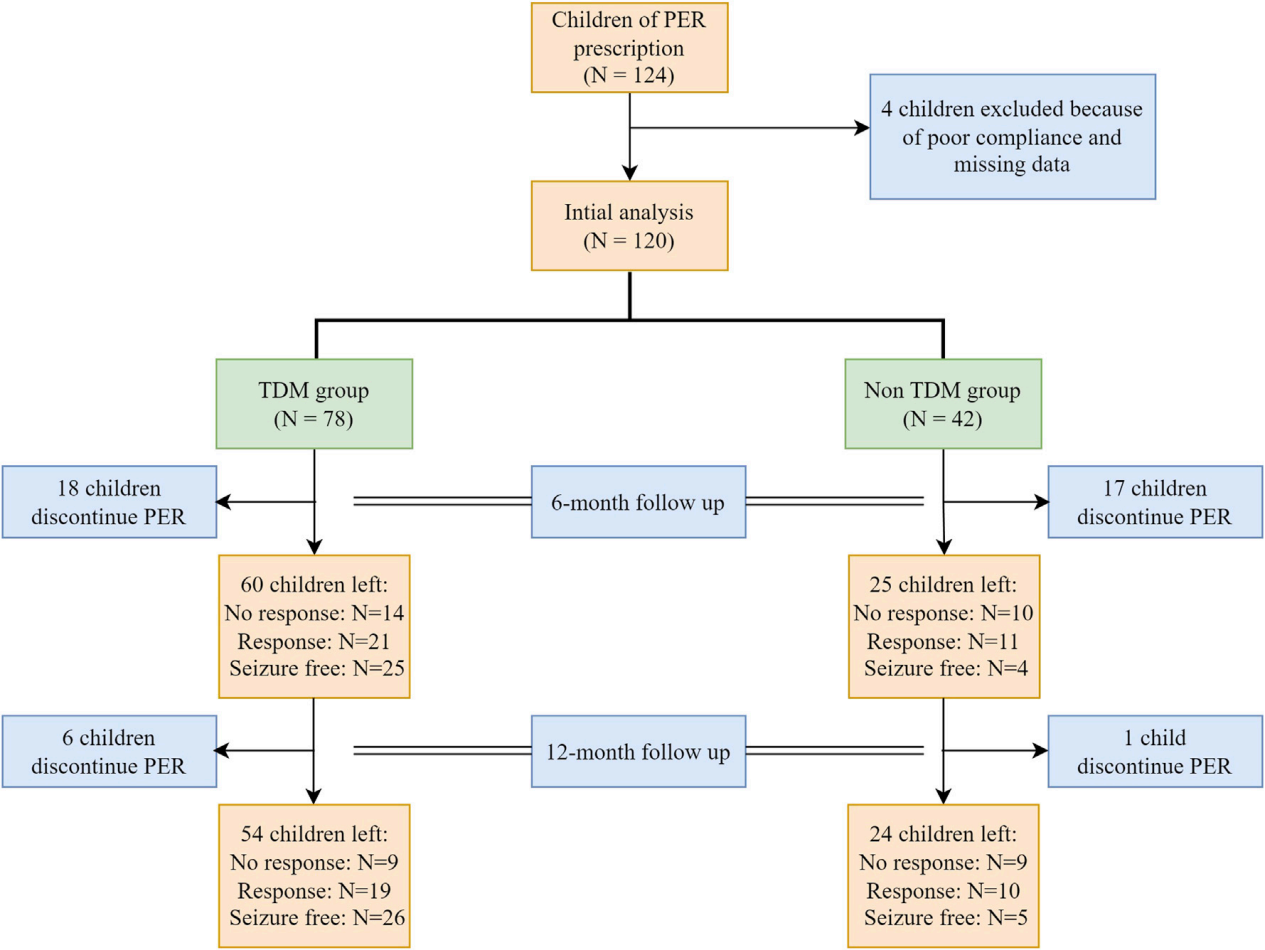
Patient flow. PER, perampanel.

Between 1 October 2020, and 30 September 2022, a total of 124 children with epilepsy were treated with PER. Four children were excluded because of poor compliance. A total of 120 patients were analyzed, with 78 patients in the TDM group and 42 patients in the non-TDM group. The baseline characteristics before PER prescription are shown in Table 1. Thirty-five patients (28 in the TDM group and 17 in the non-TDM group) discontinued PER at the 6-month follow-up. Eighty patients (54 and 24 in the TDM and non-TDM groups, respectively) underwent regular PER for 1 year and were analyzed for final clinical outcomes. The patient flowchart is shown in Figure 2. Age, sex, epilepsy type, newly diagnosed epilepsy status, family history, and average daily dose of PER were no significantly difference so that the two groups were comparable. Most patients used 2 or more kinds of ASMs before PER prescription. The most commonly used drug was VPA (40.8%), followed by LEV (29.2%). The baseline characteristics and clinical outcomes of patients who started taking PER are shown in Table 2. The average daily dose of PER in the TDM group was similar to that in the non-TDM group. More children in the TDM group were seizure free (48.2% VS. 16.7%) than those in the non-TDM group (p = 0.035). A total of 83.3% of the children responded after PER prescription, which was better than that in the non-TDM group (58.3%).

**TABLE 1:** Baseline characteristics of the children before PER prescription (n = 120).

Group	TDM	Non TDM
Number	78	42
Age, year (SD)	7.29 (4.70)	6.11 (3.86)
Male (%)	42 (53.8)	28 (66.7)
Epilepsy type		
Focal epilepsy	51	25
Generalized epilepsy	24	10
Unclassified epilepsy	3	7
Drug- resistant epilepsy	48	35
Number of ASM before PER prescription (SD)	3 (2)	4 (2)
Family history (%)	14 (17.9)	5 (11.9)
Used ASM before PER prescription		
VPA	36	13
LEV	25	10
TPM	19	8
OXC	15	4
LTG	10	2
NZP	8	5
CLZ	6	5
CLB	6	2
CBZ	4	3
ZNS	4	3
VGB	4	0
PB	2	1
LCS	1	0

LTG, lamotrigine; TPM, Topiramate; LEV, Levetiracetam; CLZ, Clonazepam; ZNS, Zonisamide; VPA, valproate; CBZ, Carbamazepine; NZP, Nitrazepam; OXC, Oxcarbazepine; LCS, lacosamide; CLB, Clobazam; VGB, Vigabatrin; PB, phenobarbital; PER, perampanel; SD, standard deviation; ASM, anti-seizure medication.

**TABLE 2:** Characteristics and clinical outcomes of children after 12 months of PER treatment (n = 78).

Group	TDM	Non TDM	P value
Number	54	24	
Age, year (SD)	6.49 (3.95)	8.64 (4.77)	0.191
Male (%)	28 (52)	17 (71)	0.117
Epilepsy type			
Focal epilepsy	37	14	0.279
Generalized epilepsy	14	6	
Unclassified epilepsy	3	4	
Drug- resistant epilepsy	30	18	0.103
Number of ASM before PER prescription (SD)	2 (2)	3 (2)	0.383
Family history (%)	9	4	1.000
Clinical outcome			
Response	19	10	0.035
Seizure free	26	4	
Uncontrolled	9	8	

PER, perampanel; SD, standard deviation; ASM, anti-seizure medication.

The cost, transition probability, and OR captured from real-world data were used as parameters to populate the Markov model. All parameters were related to health status. The transition probability of the first cycle was greater than that of the second cycle, indicating that the efficacy of the PER was better at the initial prescription stage than at later stages. The OR of seizure freedom of the non-TDM group relative to the TDM group from the Ordinal Logistic Regression was 0.461 (95% CI: 0.22–0.965, P = 0.040). We characterized the actual mean 6-month costs for the health state. The mean total costs per patient in the seizure-free state were $488.70 in the non-TDM group and $609.15 in the TDM group, which were significantly lower than those in the non-TDM group ($807.08 in the non-TDM group vs. $688.95 in the TDM group). Total costs were driven by PER costs (non-TDM: $177.93; TDM: $180.69), TDM costs, outpatient costs, hospitalization costs (non-TDM: $113.27; TDM: $351.18) and other ASM costs (non-TDM: $330.95; TDM: $222.89). Patients in the TDM group were more likely to be outpatients than those in the non-TDM group were. All the parameter inputs are shown in Table 3. The unit cost of drugs and routine monitoring were displayed in Tables 4, 5.

**TABLE 3:** Overview of cost, quality of life and clinical inputs.

Parameter	Value (sensitivity analysis range*)		Distributions	Source
	Non TDM	TDM		
OR:100% seizure reduction VS TDM	0.461 (0.220–0.965)	-	Lognormal	Real world data
Probability of adverse reaction				
Dizziness (%)	9.1 (7.3–10.9)	4.8 (3.8–5.7)	Beta	Li Y et al. (2022); Qu et al. (2023); Lin et al. (2018)
Somnolence (%)	7.6 (6.1–9.1)	1.8 (1.4–2.2)		
Irritable (%)	10.6 (8.5–12.7)	1.2 (1.0–1.4)		
Ataxia (%)	4.5 (3.6–5.5)	1.2 (1.0–1.4)		
Transition probability of 1st cycle (%)				
No response to no response	20.0%		-	Real world data
No response to response	26.7%			
No response to seizure freedom	24.1%			
No response to discontinue PER	29.2%			
Transition probability of 2nd cycle				
No response to no response	70.8%		-	Real world data
No response to response	4.2%			
No response to seizure freedom	0.0%			
No response to discontinue PER	25.0%			
Response to stop	3.1%			
Response to response	87.5%			
Response to no response	3.1%			
Response to seizure freedom	6.3%			
Seizure freedom to seizure freedom	100.0%			
Probability of combination with another drug				
Seizure free				
LTG	0.0 (0.0–20.0)	0.0 (0.0–20.0)	Beta	Real world data
TPM	20.0 (16.0–24.0)	12.8 (10.3–15.4)		
LEV	30.0 (24.0–36.0)	28.2 (22.6–33.8)		
CLZ	20.0 (16.0–24.0)	2.6 (2.1–3.1)		
ZNS	20.0 (16.0–24.0)	2.6 (2.1–3.1)		
VPA	40.0 (32.0–48.0)	38.5 (30.8–46.2)		
CBZ	20.0 (16.0–24.0)	0.0 (0.0–20.0)		
XXP	10.0 (8.0–12.0)	7.7 (6.2–9.2)		
OXC	10.0 (8.0–12.0)	23.1 (18.5–27.7)		
LCS	0.0 (0.0–20.0)	0.0 (0.0–20.0)		
CLB	0.0 (0.0–20.0)	0.0 (0.0–20.0)		
VGB	0.0 (0.0–20.0)	0.0 (0.0–20.0)		
PB	0.0 (0.0–20.0)	2.6 (2.1–3.1)		
Response				
LTG	0.0 (0.0–20.0)	13.8 (11.0–16.6)	Beta	Real world data
TPM	25.0 (20.0–30.0)	34.5 (27.6–41.4)		
LEV	43.8 (35.0–52.5)	27.6 (22.1–33.1)		
CLZ	43.8 (35.0–52.5)	10.3 (8.3–12.4)		
ZNS	6.3 (5.0–7.5)	6.9 (5.5–8.3)		
VPA	37.5 (30.0–45.0)	34.5 (27.6–41.4)		
CBZ	18.8 (15.0–22.5)	6.9 (5.5–8.3)		
XXP	18.8 (15.0–22.5)	20.7 (16.6–24.8)		
OXC	0.0 (0.0–20.0)	10.3 (8.3–12.4)		
LCS	6.3 (5.0–7.5)	3.4 (2.8–4.1)		
CLB	0.0 (0.0–20.0)	0.0 (0.0–20.0)		
VGB	6.3 (5.0–7.5)	3.4 (2.8–4.1)		
PB	6.3 (5.0–7.5)	0.0 (0.0–20.0)		
No response				
LTG	30.8 (24.6–36.9)	18.8 (15.0–22.5)	Beta	Real world data
TPM	23.1 (18.5–27.7)	56.3 (45.0–67.5)		
LEV	76.9 (61.5–92.3)	25.0 (20.0–30.0)		
CLZ	30.8 (24.6–36.9)	6.3 (5.0–7.5)		
ZNS	23.1 (18.5–27.7)	0.0 (0.0–20.0)		
VPA	46.2 (36.9–55.4)	62.5 (50.0–75.0)		
CBZ	7.7 (6.2–9.2)	0.0 (0.0–20.0)		
XXP	7.7 (6.2–9.2)	25.0 (20.0–30.0)		
OXC	7.7 (6.2–9.2)	12.5 (10.0–15.0)		
LCS	0.0 (0.0–20.0)	0.0 (0.0–20.0)		
CLB	7.7 (6.2–9.2)	6.3 (5.0–7.5)		
VGB	0.0 (0.0–20.0)	6.3 (5.0–7.5)		
PB	0.0 (0.0–20.0)	0.0 (0.0–20.0)		
The number of TDM	-	1.74 (0.64–2.83)	Gamma	Real world data
Total cost per cycle				
Seizure free	488.70	609.16	Gamma	Real world data
Response	641.99	872.10		
No response	807.08	988.95		
Discontinue PER	787.31	787.31		
Hospitalization cost				
Seizure free	99.36	179.96	Gamma	Real world data
Response	151.22	384.48		
No response	101.26	420.14		
Discontinue PER	101.26	420.14		
Outpatient cost				
Seizure free	55.75	84.66	Gamma	Real world data
Response	52.09	116.03		
No response	100.22	135.13		
Discontinue PER	100.22	135.13		
PER cost				
Seizure free	171.05	189.92	Gamma	Real world data
Response	189.98	187.35		
No response	172.76	164.80		
Other ASM cost				
Seizure free	162.54	136.52	Gamma	Real world data
Response	248.70	166.15		
No response	432.84	250.78		
Discontinue PER	479.73	338.13		
Number of outpatient				
Seizure free	2.43 (0.84–4.02)	3.83 (2.43–1.40)	Normal	Real world data
Response	2.77 (0.84–4.70)	4.97 (1.66–8.28)		
No response	4.89 (1.51–8.27)	6.40 (0.72–13.52)		
Discontinue PER	4.89 (1.51–8.27)	6.40 (0.72–13.52)		
Adverse reaction cost				
Dizziness	318.7 (224.0–596.4)		Gamma	China Health Statistics Yearbook (2022)
Somnolence	318.7 (224.0–596.4)			
Irritable	318.7 (224.0–596.4)			
Ataxia	318.7 (224.0–596.4)			
TDM cost	-	121.8	Gamma	Real world data
Utility of health state				
Seizure free	0.650 (0.520–0.780)		Beta	utility mapping study, Real world data
Response	0.597 (0.480–0.720)			
No response	0.500 (0.400–0.600)			
Discontinue PER	0.500 (0.400–0.600)			
Utility of adverse reaction				
Dizziness	−0.0047 (−0.0037–0.0057)		Gamma	Laskier et al. (2023)
Somnolence	−0.0047 (−0.0037–0.0057)			
Irritable	−0.0047 (−0.0037–0.0057)			
Ataxia	−0.0047 (−0.0037–0.0057)			

LTG, lamotrigine; TPM, Topiramate; LEV, Levetiracetam; CLZ, Clonazepam; ZNS, Zonisamide; VPA, valproate; CBZ, Carbamazepine; NZP, Nitrazepam; OXC, Oxcarbazepine; LCS, lacosamide; CLB, Clobazam; VGB, Vigabatrin; PB, phenobarbital; PER, perampanel; ASM, anti-seizure medication; TDM, therapeutic drug monitoring.

**TABLE 4:** The unit cost of ASM ($/g).

Drugs	The unit price ($/g)
CBZ	0.64
CLB	47.10
CLZ	36.85
LCS	19.53
LEV	1.88
LTG	8.50
OXC	1.84
PB	2.58
PER	335.81
TPM	7.61
VPA	0.71
XBN	5.50
NZP	4.99
ZNS	9.44

LTG, lamotrigine; TPM, Topiramate; LEV, Levetiracetam; CLZ, Clonazepam; ZNS, Zonisamide; VPA, valproate; CBZ, Carbamazepine; NZP, Nitrazepam; OXC, Oxcarbazepine; LCS, lacosamide; CLB, Clobazam; VGB, Vigabatrin; PB, phenobarbital; PER, perampanel; ASM, anti-seizure medication.

**TABLE 5:** Unit cost of hospitalization and outpatient.

Group	TDM			Non-TDM		
	Seizure free	Response	No response	Seizure free	Response	No response
Probability of Hospitalization	43.1%	47.5%	47.5%	20.0%	23.1%	16.7%
Numbers of Hospitalization	1.09	1.63	1.18	1.00	1.00	1.00
Days of Hospitalization	1.75	2.95	3.60	3.48	6.48	3.44
Average Daily Cost of Hospitalization ($)	219.40	295.95	206.05	142.76	101.46	173.15
Total Cost of Hospitalization ($)	179.96	384.48	420.14	99.36	151.22	101.26
Number of Outpatient	3.83	4.97	6.40	2.43	2.77	4.89
Average Cost of Consultation ($)	10.82	12.06	9.83	11.66	7.52	9.21
Average Cost of Test ($)	24.90					
Average Cost of Examination ($)	22.55					

### Cost effectiveness analysis

Compared to non-TDM, TDM of PER had an ICER of $732.90 per QALY gained by increasing costs from $12275.89 to $12590.69 and increasing QALYs from 8.66 to 9.09, which was significantly lower than the $12814 per QALY gained, showing that TDM of PER was highly cost-effective in pediatric patients with epilepsy (Table 6).

**TABLE 6:** Costs and effectiveness of TDM in children with epilepsy.

Analysis	Cost ($)			QALYS			ICER ($/QALYS)
	Non TDM	TDM	Incremental	Non TDM	TDM	Incremental	
Base case	12,275.89	12,590.69	314.80	8.66	9.09	0.43	732.91
Newly diagnosed epilepsy	12,448.09	10,862.02	(1,586.07)	8.85	9.71	0.86	Dominant
Refractory epilepsy	11,775.38	13,363.39	1,588.01	8.56	8.80	0.24	6,696.31
1-year TDM	12,189.19	12,611.53	422.34	8.67	9.08	0.42	1,012.30
1 year time horizon	2,224.35	2,484.55	260.20	1.48	1.54	0.06	4,112.92
5-year time horizon	6,614.82	6,964.91	350.08	4.59	4.83	0.24	1,480.36
10-year time horizon	10,121.84	10,464.10	342.25	7.12	7.47	0.36	957.49

Notes. red parentheses represent negative numbers.

**FIGURE 3:**
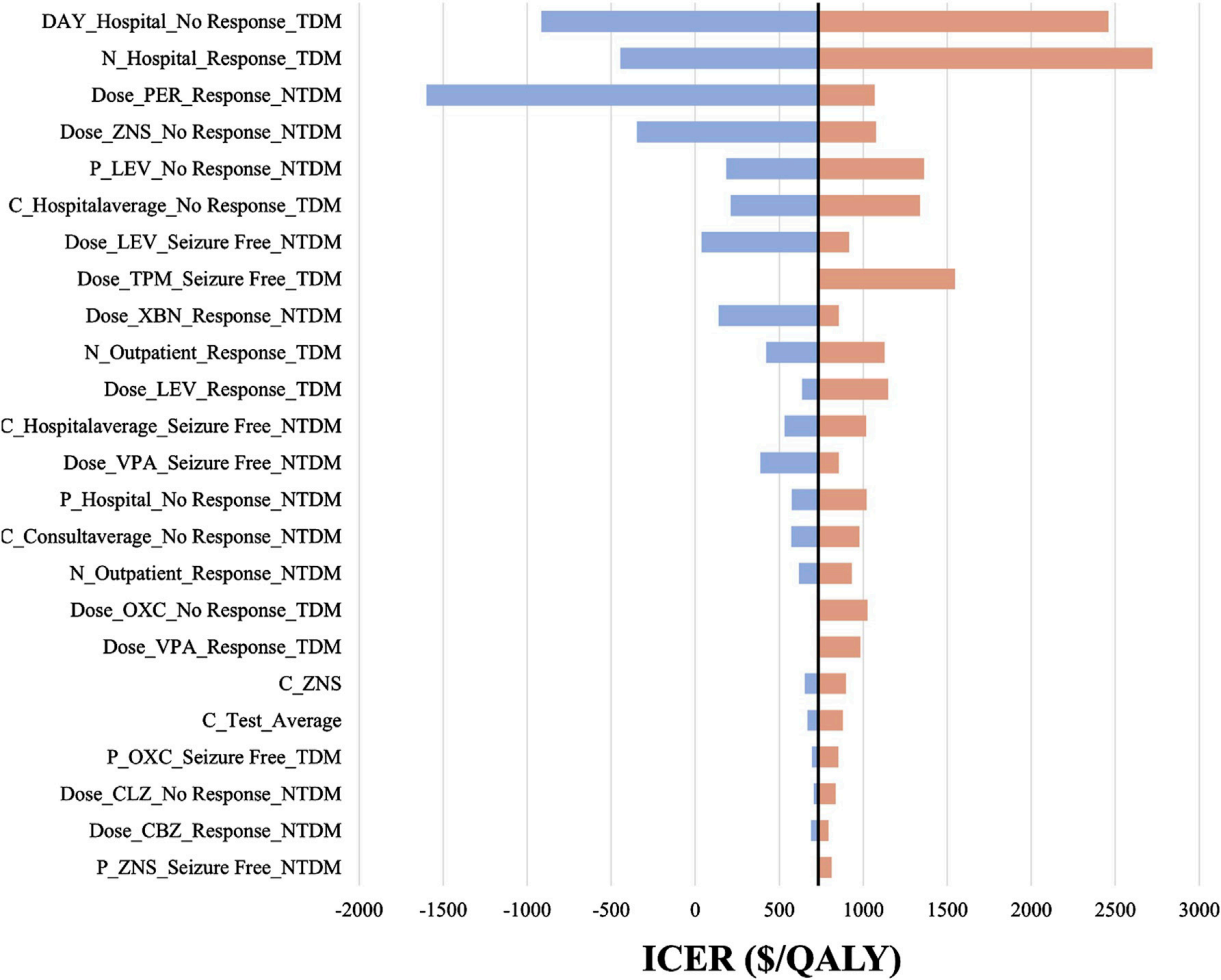
The tornado diagram. The black straight line represents the willingness to pay of $732.90 per QALY gained. P represents probability, N represents number, C represents cost, “_” represents of or in, Hospital represents hospitalization, NTDM represents Non-TDM group. For example, C_Hospitalaverage_Seizure Free_NTDM means the average daily cost of hospitalization in “Seizure Free” health state in Non-TDM group. LEV, Levetiracetam; CLZ, Clonazepam; ZNS, Zonisamide; VPA, Valproate; CBZ, Carbamazepine; OXC, Oxcarbazepine; PER, Perampanel.

**FIGURE 4:**
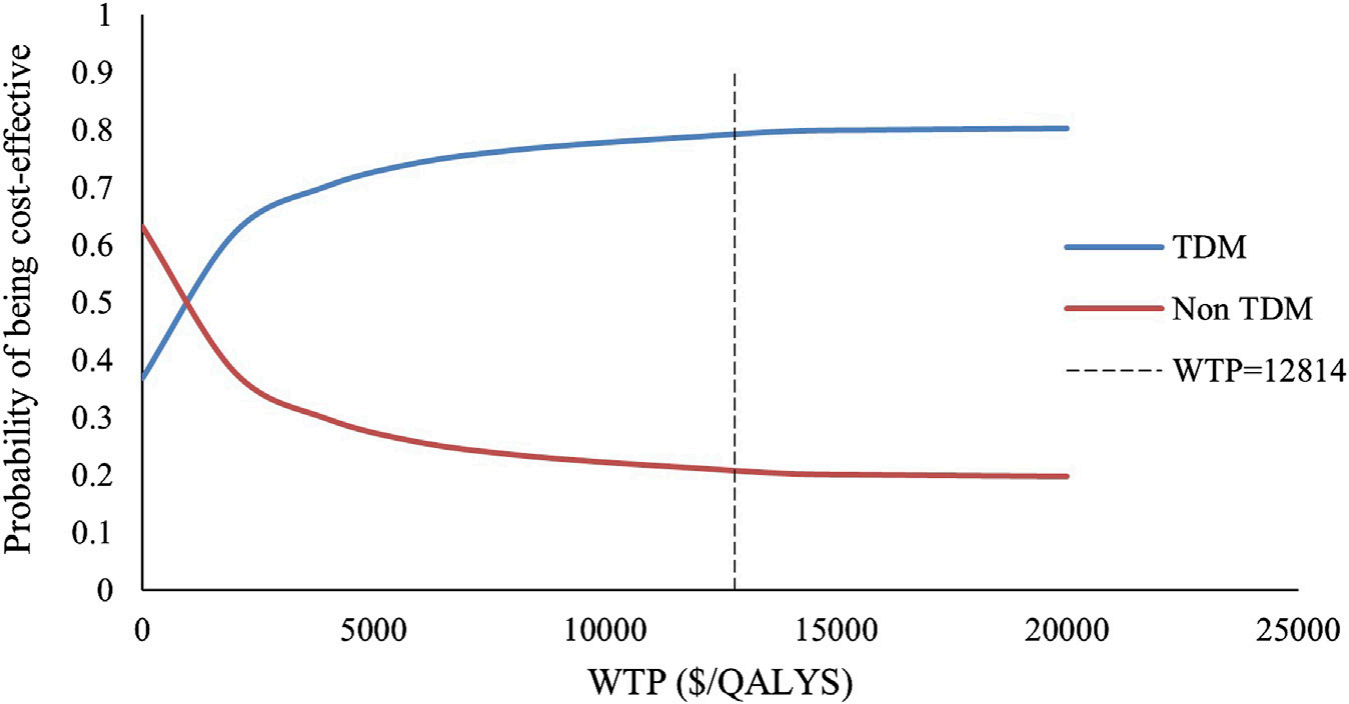
The acceptable probability curve of WTP.

We included 240 influencing factors in the one-way sensitivity analysis and showed that days of hospitalization in the no response state and the number of hospitalizations in the response state related to the cost of hospitalization were the largest influencing factors. Figure 3 shows the factors that impact the results. Regardless of the effect of these factors on the baseline ICER, TDM is always highly cost-effective relative to non-TDM. The probability sensitivity analysis revealed that TDM of the PER had a 79.11% probability of being highly cost-effective (Figure 4).

The scenario analysis results are shown in Table 6. When TDM was restricted to patients with newly diagnosed epilepsy who received no other ASM, TDM was dominant, with an increased QALY of 0.86 and a decreased cost of $1,586.07. When TDM was restricted to drug-resistant epilepsy, the resulting ICER was $6,696.31 per QALY gained. Compared with continuous TDM in base case analysis, 1-year TDM had lower costs but resulted in more adverse reactions. The resulting ICER for 1-year TDM compared with the non-TDM group was $1,012.30 per QALY gained. TDM was highly cost effective over 1-, 5- and 10-year time horizons ($4,112.92, $1,480.36 and $957.49/QALY, respectively).

## Discussion

Among children with epilepsy receiving PER, TDM was more effective than PER without TDM in improving seizure control. Additionally, TDM was highly cost-effective at a WTP of $12,814 per QALY gained. This finding was consistent across the one-way sensitivity analysis and scenario analysis. In children with newly diagnosed epilepsy first prescribed PER, TDM was strongly dominant.

Most studies that have focused on the TDM of PER have reported the relationship between its effectiveness and the plasma or saliva concentration (Li Y. et al., 2022; Kim et al., 2020; Yamamoto et al., 2017). However, few studies have explored the utilization of TDM in clinical settings and the impact of dosage adjustment based on TDM results on clinical outcomes. Only one study described 68 patients who received TDM due to poor seizure control but did not describe the subsequent improvement in seizure control (Lin et al., 2024). Our study is the first to report the clinical application of TDM of PER, comparing the clinical outcome between TDM and without TDM after PER prescription, which reflects the real-world use of TDM in children with epilepsy. We found that the application of TDM to PER significantly improved the clinical outcomes of children with epilepsy, increasing the 1-year seizure-free rate from 16.7% to 48.1% and decreasing the seizure frequency >50%, from 58.3% to 83.3%. The positive impact of TDM of PER on clinical outcomes may be related to factors such as the combined use of drugs affecting free concentrations (Qu et al., 2023; Yu et al., 2023) and achieving suitable maintenance doses more quickly.

Our study illustrated the economic value of TDM of the PER in children with epilepsy. We usually evaluate drugs comprehensively based on their safety, effectiveness, cost-effectiveness, affordability, innovation, suitability, and accessibility, as does TDM. A study conducted in Malaysia showed that serum level monitoring of ASMs is cost-effective, as the ICER is MRY 29,666 per patient with 3 months of seizure freedom (Salih et al., 2013). Forty-two percent of the TDM was VPA, followed by CBZ, phenytoin sodium, and PB, accounting for 38%, 12%, and 8%, respectively. With the increasing use of new ASMs, further research needs to be conducted on the TDM of PER and other new ASMs. Our study combined the Markov model and real-world data and revealed that TDM of the PER is cost-effective in children with epilepsy and deserves widespread use in the clinical setting. The sensitivity analysis confirmed that the therapeutic advantages of TDM compared to non-TDM do not impact the cost-effectiveness.

Since the findings are derived from real-world data, they come with both pros and cons. The upside is that they better reflect actual conditions, whereas the downside is the presence of confounding variables, like disease severity, that may affect the results. As refractory epilepsy has greater costs and a lower remission rate than newly diagnosed epilepsy (Begley and Durgin, 2015), we conducted a cost-effectiveness analysis to determine the sensitivity of the results by dividing patients into newly diagnosed epilepsy and refractory epilepsy groups. We found that TDM strongly dominated in children with epilepsy who received PER as initial treatment. Approximately 70% of patients with newly diagnosed epilepsy can reach seizure-free status after initial drug treatment, and 30% will develop refractory epilepsy. As the number of ASMs increases, the remission rate decreases (Chen et al., 2018). Therefore, it is crucial to achieve seizure-free status for newly diagnosed epilepsy patients at initial treatment, as TDM can increase the remission rate and reduce costs. In refractory epilepsy, the remission rate is usually very low, and the improvement of the remission rate by TDM is also very limited.

Common adverse reactions of PER include dizziness, irritability, sleepiness, and in severe cases, may lead to the discontinuation of PER in patients (D'Aniello et al., 2025). TDM can help reduce these side effects to some degree. We based our analysis on the incidence of adverse reactions in epilepsy patients undergoing TDM from prior studies (Li D. et al., 2022; Rugg-Gunn, 2014). Those studies didn't report psychiatric side effects linked to PER, and we didn't include them in our research either. This might be because psychiatric and behavioral side effects are rare in pediatric patients (1.5%) (Mammì et al., 2022), and weren't observed in the populations of previous studies. Thus, we can’t confirm if TDM can mitigate PER’s impact on neuropsychiatric side effects, suggesting the need to include a systematic monitoring of neurobehavioral symptoms in the context of TDM.

The reference range we used to interpret TDM results and guide dose adjustment was 100–1000 ng/mL. In the USA and Japan, a therapeutic range of 180–980 ng/mL is applied to patients aged >12 years old (Yamamoto et al., 2017; Gidal et al., 2014; Patsalos, 2015), while in Chinese patients aged 0–18 years old, it’s 180–610 ng/mL (Li Y. et al., 2022) or 100–1000 ng/mL (Yu et al., 2023). The therapeutic range for PER varies across countries. Future studies should focus more on the relationship between efficacy and therapeutic range for specific age groups.

There are limitations in our study. First, we used retrospective data, and some confounding factors could not be captured, such as genotype, adverse reactions, etiology of epilepsy, and the reason why TDM. This restricted our ability to analyze individual differences in response to TDM and introduce selection bias. Second, the utilities of pediatrics were not directly from real world data. Instead, they were estimated by mapping variables such as seizure frequency from SF-36, which may affect the results. However, there were few studies on TDM related quality of life in pediatrics with epilepsy. Future studies should address this gap. Third, a small and imbalanced sample size may introduce bias and impact the results. In addition, cost-effectiveness estimates for TDM of PER may not be generalizable to other ASMs or adults because the clearance of PER varies at different ages, and the effect of TDM for other ASMs may vary, especially for ASMs with multiple drug interactions (Patsalos et al., 2018). However, the results and analysis could provide a useful framework to guide the clinical use of TDM on a larger scale.

## Conclusion

TDM of PER could reduce seizure frequency and is cost-effective for children with epilepsy. TDM of the PER in newly diagnosed epilepsy patients is strongly dominant because of its improvement in efficacy and reduction in cost.

## Data Availability

The original contributions presented in the study are included in the article/supplementary material, further inquiries can be directed to the corresponding authors.
